# “Screened Out but Not Referred In”: The Unmeasured Psychiatric Referral Gap for Body Dysmorphic Disorder in Cosmetic Practices

**DOI:** 10.1192/bjo.2026.11111

**Published:** 2026-06-30

**Authors:** Augusta Okoro

**Affiliations:** St Andrew’s Healthcare, Northampton, United Kingdom

## Abstract

**Aims:**

Body Dysmorphic Disorder (BDD) is overrepresented in aesthetic medicine (prevalence up to 18.6% in aesthetic cohorts versus ~1.7–2.9% in the general population). Professional bodies recommend psychological assessment before cosmetic intervention and referral to mental health services if BDD is suspected.

We aim to examine post-assessment care by:

• Quantifying the gap between recommended assessment/referral standards and real-world practice.

• Assessing how well the literature captures referral conversion (screening to specialist assessment).

• Identifying patient, clinician, and system barriers that prevent completion of specialist mental-health assessment.

**Methods:**

A targeted review was conducted using evidence from aesthetic-surgery meta-analyses, dermatology and plastic surgery guidelines, aesthetic-practice safety documents, insurer psychological-risk frameworks, and psychiatric assessment resources. Sources were reviewed for prevalence data, referral expectations and realities, and documented barriers to mental-health engagement.

**Results:**

Although BDD prevalence in aesthetic settings is consistently studied and reported,published literature rarely captures how many screen-positive patients are referred for psychiatric assessment.

Despite the presence of several high-accuracy validated BDD screening tools, recent database evidence highlights ongoing missed detection: in a cohort of 226,374 cosmetic-surgery patients (from August 2002–August 2022), 52.1% of BDD diagnoses occurred only after procedures.

Literature shows that barriers span patient-level (denial, doctor-shopping), clinician-level (concerns about conflict, reputational or financial impact, limited training), and system-level (lack of structured referral pathways, limited multidisciplinary integration, absence of warm-handover mechanisms). However, there are minimal quantitative studies measuring these factors.

UK-based literature documents BDD prevalence and outcome risk in cosmetic seekers and provides validated screening tools and professional standards, but there is minimal evidence reporting UK metrics on referral-conversion or psychiatric follow-through.

**Conclusion:**

Professional guidelines recommend that suspected BDD requires specialist evaluation, yet referral completion rates remain unmeasured and pathway implementation inconsistent across aesthetic practice settings. The absence of referral-conversion data and evidence of missed diagnoses, highlight a critical knowledge and safety gap. Priority actions include: auditing current screening and referral practices in regulated aesthetic settings, establishing clear referral triggers and destinations, implementing warm-handover mechanisms, and developing integrated pathways between aesthetic and mental health services to improve early detection and patient safety.

